# Evaluation of a Novel Web-Based Active Learning Tool for Primary Care Physicians’ Continuing Professional Development (The Community Fracture Capture Learning Hub): Quantitative Analysis

**DOI:** 10.2196/76216

**Published:** 2026-01-21

**Authors:** Ahmed M Fathalla, Cherie Chiang, Ralph Audehm, Alexandra Gorelik, Shanton Chang, Thang Dao, Christopher J Yates, Steve Snow, Rahul D Barmanray, Sarah Price, Lucy Collins, John D Wark

**Affiliations:** 1 Department of Medicine, The Royal Melbourne Hospital, University of Melbourne, 300 Grattan Street, Parkville, Melbourne, 3050, Australia, +61 03 8344 5892; 2 Department of Diabetes & Endocrinology, Royal Melbourne Hospital, Melbourne Health, Melbourne, Australia; 3 Department of General Practice and Primary Care, University of Melbourne, Melbourne, Australia; 4 School of Public Health and Preventive Medicine, Monash University, Melbourne, Australia; 5 School of Computing and Information Systems, University of Melbourne, Melbourne, Australia; 6 Praxhub, Melbourne, Australia; 7 Department of Obstetric Medicine, Royal Women's Hospital, University of Melbourne, Melbourne, Australia; 8 Department of Medicine, School of Clinical Sciences, Monash University, Melbourne, Australia

**Keywords:** case-based education, CFC, community-based fracture capture learning hub, continuing professional development, CPD, online learning platform, osteoporosis, PCPs, primary care physicians, VCoP, virtual communities of practice

## Abstract

**Background:**

The lack of osteoporosis treatment initiation following fragility fractures is a recognized gap, particularly in primary care. Primary care physicians’ (PCPs) barriers to treatment, such as uncertainties in investigation, initiation, and concerns about drug side effects, remain challenging. It is also unclear whether knowledge gaps and barriers vary by region or if active learning platforms are more effective than passive methods in improving treatment rates, and how PCP demographics influence learning outcomes. With time constraints, PCPs are increasingly using online platforms for continuing professional development, and the interactive online Community Fracture Capture (CFC) tool has emerged as a promising alternative to traditional methods. Our CFC pilot study tested this program’s design and content, revealing its potential effectiveness.

**Objective:**

The study aimed to assess the operational characteristics, educational effectiveness, and acceptability of the interactive online CFC model in enhancing Australian PCPs’ knowledge and skills in community-based fracture treatment. Additionally, it sought to examine how PCPs’ knowledge and treatment gaps relate to their demographic characteristics and clinical practice backgrounds.

**Methods:**

The CFC Learning Hub is a secure, adaptable online platform that promotes community learning. It includes an interactive forum where participants share case studies and engage in discussions with bone specialists and senior PCP facilitators. The hub also offers a knowledge repository and allows participants to post inquiries. Online surveys and back-end analytics track baseline knowledge, activity levels, and improvements in knowledge and confidence over time, offering insights into participants’ learning and program development.

**Results:**

Four 6-week small-group cycles involved 55 PCPs, with over 80% working in metropolitan-based practices and a median (IQR) of 22 (16-34) years in practice. Topic modules covered osteoporosis diagnostics, treatment, monitoring, and challenging conditions, using a multidisciplinary approach with participant case studies. A total of 35 (64%) PCPs provided evaluation data, with 86% (n=30) joining to learn from experts or improve patient management and 83% (n=29) being satisfied with the content. Preferred learning methods included small group learning (n=13, 37%), live webinars (n=9, 26%), interactive learning (n=7, 20%), and on-demand videos (n=6, 17%), and 57% (n=20) found the platform easy to use. The most popular access times were evening (n= 23, 66%) and weekends (n=10, 29%). At completion, 89% (n=31) would recommend the training, and 78% (n=22 out of 28 respondents to the postprogram expectations meeting survey) were fully satisfied that their training needs were met, with 22% (n=6) partly satisfied. In addition, following the course completion, almost everyone reported being confident or very confident in managing osteoporosis.

**Conclusions:**

The CFC program was created by bone specialists, PCPs, software engineers, and information technology specialists. This collaboration produced a user-friendly, case-based, interactive, time-flexible, and highly acceptable program bridging investigation and management gaps in osteoporosis. It is customized to address challenges faced by PCPs and is potentially relevant for implementation in a wide range of fields, both health-related and others.

## Introduction

### Addressing the Global Challenge of Osteoporosis: Investigation and Treatment Gaps

Osteoporosis poses a significant global health concern, affecting approximately 500 million individuals worldwide, and is associated with 37 million fragility fractures annually in people aged over 55 years [1,2]. This burden is expected to escalate with the aging global population, with projections indicating a 310% increase in hip fractures for men and a 240% increase for women by 2050 [3,4]. Despite this increasing prevalence of osteoporosis and the presence of effective diagnostic tools, fracture risk assessment tools (eg, Fracture Risk Assessment Tools and dual-energy X-ray absorptiometry scanning), and appropriate medications, there persists a treatment gap, with only 20% to 30% of patients with fragility fracture receiving guidelines-based care [4,5]. For instance, a study of 145,185 Medicare-enrolled patients with fragility fractures in the United States found that only 30.4% of the cohort received an osteoporosis treatment in the 12-month postfracture period [6].

In Australia, a societal cost of Aus $2.6 billion (approximately US $1.7 billion) was attributable to osteoporosis in 2022 alone [4]. Australian and international hospital–based fracture liaison services (FLS) have shown cost-effectiveness in secondary fracture prevention [7,8], successfully addressing this treatment gap among patients presenting with low-trauma fractures. Yet, a relative scarcity of Australian FLS centers was noted in the International Osteoporosis Foundation Capture the Fracture’s recent survey [4]. Furthermore, patients experiencing low-trauma fractures, such as those involving the vertebra or radius, which may not necessitate tertiary care, are typically assessed and treated exclusively or mainly within primary care settings, potentially bypassing hospital-based services [9]. Similar to that observed in tertiary care, primary care also faces a challenging investigation gap in primary fracture prevention, as evidenced by the plateau in bone density requests among Australian older adults despite an aging population [7,9-11].

### Enhancing Fracture Prevention: Challenges at the Primary Care Level

Adapting the hospital-based model of care to the community level presents a rational approach to enhancing fracture prevention outcomes. However, different from the hospital-based FLSs, which rely on coordinators to manage and oversee their functions, community-based fracture capture places the responsibility solely on primary care physicians (PCPs) to diagnose, examine, and treat osteoporosis [12]. Despite the availability of effective treatments and guidelines, the translation of evidence into clinical practice, particularly by PCPs, remains low in Australia, resulting in suboptimal care for many Australians eligible for osteoporosis-related investigations and therapies [5,13]. While hospital FLSs prioritize secondary fracture prevention, PCPs should additionally emphasize primary fracture prevention to prevent the first fracture [14].

### Navigating Challenges: Harnessing Virtual Communities for Professional Development in Health Care

PCPs encounter substantial challenges in managing various chronic health conditions and their associated complications within limited consultation times. Despite these constraints, PCPs must continually expand their knowledge of evidence-based practices [15,16]. Seeking online resources for continuing professional development (CPD) has become increasingly common among PCPs [17-19], offering a flexible alternative to traditional learning methods [20]. Therefore, using social media tools for CPD has emerged as a popular approach, facilitating collaborative learning in online group settings [18,21]. Virtual communities of practices (VCoPs) are among the web-based tools gaining traction in health care [22,23], particularly as their relevance became more prominent during the COVID-19 pandemic [24]. VCoPs offer health care professionals unrestricted opportunities for learning, collaboration, and information sharing, overcoming barriers like geography, cost, and time constraints [21,25,26]. Participation of health professionals in VCoPs has been associated with the alleviation of professional isolation, potentially improving program retention, fostering interprofessional collaboration, and providing a risk-free environment for active participation [27,28].

### Bridging Gaps: Exploring the Role of Virtual Communities in Addressing Osteoporosis Care Challenges

Despite the potential benefits of VCoPs to optimize health professionals’ knowledge, confidence, and practice, only a handful of recent studies, including our Community Fracture Capture (CFC) Learning Hub pilot study, have explored the effectiveness of internet-based learning activities in addressing the osteoporosis care crisis, facilitating education, maximizing health care resources, and enabling practitioners to deliver more accessible osteoporosis care at reduced costs [29,30]. Our exploration of the hurdles encountered by PCPs in using VCoPs for continuing medical education underscores the significance of fostering trust, effective time management, and collaborative learning among health care professionals to establish successful VCoPs [31]. The CFC Learning Hub program integrates flexible, asynchronous participation with a structured, interactive case-based introductory format for each topic, encouraging information to be directly linked with participants’ clinical experience. Unlike conventional lectures or scheduled webinars [32], it allows participants to engage at their own pace, accommodating clinical and personal commitments. Through continuous access to peer-to-peer and PCP-to-specialist interaction, the platform adopts collaborative knowledge exchange and critical reflection rather than unidirectional instruction and supports deeper cognitive engagement and a more nuanced understanding of clinical concepts [33]. The program’s distinctive combination of flexibility, interactivity, and collaboration differentiates it from traditional educational models and underpins the rationale for its formal evaluation in terms of operational performance, educational effectiveness, and professional impact.

Consequently, our goal is to tackle the community osteoporosis treatment gap by introducing a novel, interactive CFC learning hub, designed to create a health professionals’ VCoP that aims to facilitate knowledge transfer and overcome obstacles in osteoporosis recognition and management. We hypothesize that a CFC Learning Hub can offer a unique peer-to-peer learning platform, delivering current information on primary and secondary fracture prevention to PCPs across various regions, demographics, and experience levels. Additionally, we propose that the enhanced confidence and motivation in osteoporosis investigation and treatment through interactive web-based discussions among PCPs and expert facilitators can be quantified using analytics tools.

The primary objective of this study was to evaluate the operational characteristics and educational effectiveness of the interactive online CFC Learning Hub model in offering PCPs professional training and skills related to community fracture−related care. Secondary objectives were to (1) examine associations between PCPs’ demographic and practice backgrounds and their learning outcomes; (2) assess engagement in CPD through VCoPs and the impact of engagement levels variations on learning and knowledge acquisition; (3) describe the performance features of a tailored, interactive, case-based learning hub designed to enhance PCPs’ osteoporosis management; and (4) obtain feedback from PCP participants on the acceptability of the program.

## Methods

### Study Design and Participant Recruitment

The CFC Learning Hub is a secure and versatile online platform designed to cultivate a learning community. It includes an interactive Discussion Forum where participants share case studies and engage in guided discussions led by bone specialists and senior PCP facilitators to achieve learning goals. Moreover, the hub incorporates a robust knowledge repository and allows participants to post queries and comments. Using online surveys and back-end analytics, the platform measures baseline preactivity knowledge, monitors activity levels, and assesses progress, offering valuable insights into participants’ learning experiences and enhancements in knowledge following course completion.

Based on the literature, a relatively small group of 12 to 20 participants in a VCoP fosters greater motivation for active engagement and knowledge seeking [34,35]. Therefore, we aimed to enroll 12 to 16 PCPs per CFC Learning Hub cycle.

Participants were recruited via Praxhub, a web-based medical education platform, with electronic consent for data use after deidentification [36]. Invitations were sent through professional bodies and the platform’s internal registry. Interested PCPs were contacted by the project manager, who screened for eligibility and enrolled those who met the criteria, consented to participate in the 6-week program, and approved data use for auditing and research. Recruitment ran from May 2022 to August 2023. To foster engagement and apply clinical practicality, participating PCPs were asked to submit their own anonymized case studies, which were discussed throughout the program. We used Praxhub, an Australian-based web and mobile platform that provides free access to CPD resources and facilitates medical education for health care professionals worldwide [36]. Within customized private groups, participating PCPs accessed presentations, case studies, and discussion forums, engaged with peers and facilitators, and shared multimedia content to enhance collaboration and professional development. Comprehensive information on the study protocol has been published recently in Fathalla et al [33].

The Learning Hub explored various critical aspects of osteoporosis management through detailed topic modules. These topic modules were designed to cover osteoporosis etiology, diagnosis, its treatment and monitoring strategies, and the management of challenging conditions associated with the disorder.

### Ethical Considerations

The Melbourne Health Human Research Ethics Committee approved this project (site reference 2016.24), adhering to institutional ethical review processes for research involving human participants. Electronic informed consent from PCP participants who joined the CFC Learning Hub was obtained to use their deidentified data for research and auditing purposes. The electronic consent process included a waiver of consent for case study patients, whose anonymized information was also used in the project. The data collection and management of electronic consent were carried out using Praxhub tools [36], ensuring appropriate safeguards for privacy and confidentiality. The data used in this study were fully anonymized to protect participant identities. No financial compensation was provided to participants in this program.

## Results

### Participant Demographics and Engagement in Practice Experience Evaluation

Four 6-week small-group cycles involved 55 PCPs (total number of enrolled PCPs, N_total_=55), with 80% (44/55) of the participants practicing in metropolitan areas, and the median (IQR) years of practice being 22 (16-34) years. Of the 55 participants (N_total_), 35 PCPs (N_eval_; 64%) actively participated in the end-of-cycle evaluation process. There were no demographic or clinical practice differences between those who participated in the evaluation study and those who did not (data not shown).

The results show that those in cycles 3 and 4 had fewer years in practice (*P*=.005), were less active with a lower number of activities or clicks (*P*=.001), and accessed fewer resources (*P*=.002) compared with those in the first two cycles. Those in cycle 3 participated in less education activities (*P*=.03) and spent less time on the platform (*P*=.03) compared to those in cycles 1, 2, and 4. The weekly attendance ranged from 50% to 76%, and 61% attended 5 weeks or more.

Overall, participants were active on the platform: median (IQR) number of activities recorded via platform analytics was 42 (14-87), and all participants accessed at least 1 resource (median 6, IQR 3-10; range 1‐15; Table 1).

**Table 1.** Demographic characteristics and participation data of the study cohort^a^.

Characteristic	Cycle 1 (n=11)	Cycle 2 (n=14)	Cycle 3 (n=11)	Cycle 4 (n=19)
Years in practice, median (IQR)	25 (22‐44)	26 (19‐33)	16 (10‐22)	15 (11‐27)
Total number of different education activities^b^, median (IQR)	8 (4-14)	9 (4-13)	4.5 (2-9)	7 (5-11)
Number of resources^c^ accessed, median (IQR)	9 (6-11)	12 (4‐13)	2 (2-4)	5 (2-8)
Total number of various activities^d^, median (IQR)	85 (40-166)	91.5 (30-133)	15 (5‐28)	43 (11‐55)
Time spent on each activity (min), median (IQR)	22.5 (3.6‐76.9)	26 (6.8‐200.2)	9.5 (2.1‐55.4)	31.4 (4.7‐97.4)

a A full demonstration of resources, education activities, and various activities can be found in the program protocol [33].

b Education activities are learning activities undertaken by participants including the activities of access to case studies, knowledge hub, and educational webinar.

c Resources refer to learning resources of the program such as Osteoporosis Australia Clinical Guidelines (OACG), dietary approach for bone health, and case studies.

d Various activities refer to the group of activities conducted by participants such as provision of comments, view resources, and view education summary.

### Access Patterns and Motivations in Osteoporosis Management Program

The survey results indicate that the evening was the most popular access time for participants, with 23 of the 35 (66%) respondents opting for this time, followed by 10 of the 35 (29%) participants choosing weekends as their preferred access time, and a smaller proportion, 2 of the 35 individuals, preferred accessing it before and during working hours (Table 2). This distribution is supported by the back-end user log showing that 61% of activities were conducted during the evening or early morning hours (4 PM to 2 AM) and that 28% of activities took place on weekends (Saturday to Sunday).

Regarding reasons indicated for joining the program, 24 of the 35 (69%) individuals indicated a desire to improve patient management as their primary motivation. Additionally, 8 of the 35 (23%) participants wanted to learn from experts, while 3 of the 35 (9%) participants had other reasons for joining the program.

**Table 2.** Posteducation feedback.

Feedback	Overall, n (%)	Cycle 1, n (%)	Cycle 2, n (%)	Cycle 3, n (%)	Cycle 4, n (%)
Platform use					
Very easy or easy	20 (57)	3 (30)	6 (60)	2 (67)	9 (75)
Neutral	4 (11)	3 (30)	1 (10)	0 (0)	0 (0)
Some difficulties or very difficult	11 (31)	4 (40)	3 (30)	1 (33)	3 (25)
Profile setup					
Very easy	11 (31)	2 (20)	5 (50)	0 (0)	4 (33)
Easy	14 (40)	5 (50)	3 (30)	2 (67)	4 (33)
Neutral	7 (20)	3 (30)	2 (20)	1 (33)	1 (8)
Some difficulties	3 (9)	0 (0)	0 (0)	0 (0)	3 (25)
Motivation for participation					
Earn CPD^a^ points	2 (5.7)	2 (20)	0 (0)	0 (0)	0 (0)
Improve the management or treatment of patients	24 (69)	6 (60)	8 (80)	2 (67)	8 (67)
Learn directly from specialists	6 (17.1)	2 (20)	1 (10)	1 (33)	2 (17)
Learn from other PCPs^b^ or peers	2 (5.7)	0 (0)	0 (0)	0 (0)	2 (17)
Other	1 (2.9)	0 (0)	1 (10)	0 (0)	0 (0)
Best time					
After work	23 (66)	7 (70)	5 (50)	2 (67)	9 (75)
Before work	1 (3)	0 (0)	1 (10)	0 (0)	0 (0)
During work hours	1 (3)	0 (0)	1 (10)	0 (0)	0 (0)
Weekends	10 (28.6)	3 (30)	3 (30)	1 (33)	3 (25)
Device					
Desktop or laptop	23 (66)	6 (60)	8 (80)	1 (33)	8 (67)
Smartphone	7 (20)	2 (20)	1 (10)	1 (33)	3 (25)
Tablet	5 (14)	2 (20)	1 (10)	1 (33)	1 (8)
Group size					
The right size	29 (83)	9 (90)	7 (70)	3 (100)	10 (83)
Too few	5 (14)	1 (10)	3 (30)	0 (0)	1 (8)
Too many	1 (3)	0 (0)	0 (0)	0 (0)	1 (8)
Total facilitators					
1 PCP and 1 specialist	29 (83)	8 (80)	9 (90)	2 (67)	10 (83)
1 PCP	4 (11)	1 (10)	1 (10)	1 (33)	1 (8)
1 Specialist	2 (6)	1 (10)	0 (0)	0 (0)	1 (8)
Satisfied with content					
Very satisfied	19 (54)	3 (30)	9 (90)	1 (33)	6 (50)
Satisfied	10 (29)	5 (50)	0 (0)	1 (33)	4 (33)
Neutral	5 (14)	2 (20)	1 (10)	1 (33)	1 (8)
Unsatisfied	1 (3)	0 (0)	0 (0)	0 (0)	1 (8)
Satisfied overall					
Very satisfied	15 (43)	3 (30)	7 (70)	1 (33)	4 (33)
Satisfied	17 (49)	6 (60)	3 (30)	2 (67)	6 (50)
Neutral	2 (6)	1 (10)	0 (0)	0 (0)	1 (8)
Unsatisfied	1 (2.9)	0 (0)	0 (0)	0 (0)	1 (8)
Preferred learning style					
Interactive learning	7 (20)	1 (10)	2 (20)	0 (0)	4 (33)
Live webinar	9 (26)	0 (0)	3 (30)	1 (33)	5 (42)
On-demand video	6 (17)	4 (40)	1 (10)	0 (0)	1 (8)
Small group learning	13 (37)	5 (50)	4 (40)	2 (67)	2 (17)
How likely are you to recommend the training					
Very likely	17 (49)	3 (30)	7 (70)	1 (33)	6 (50)
Likely	14 (40)	6 (60)	2 (20)	2 (67)	4 (33)
Neutral	2 (6)	1 (10)	1 (10)	0 (0)	0 (0)
Unlikely	1 (3)	0 (0)	0 (0)	0 (0)	1 (8)
Very unlikely	1 (3)	0 (0)	0 (0)	0 (0)	1 (8)

a CPD: continuing professional development.

b PCP: primary care physician.

### Participant Preferred Model of Learning

The study’s findings revealed diverse preferences among participants regarding their preferred model of learning, with 37% (n=13) favoring small group learning, followed by live webinars (n=9, 26%), interactive learning (n=7, 20%), and on-demand videos (n=6, 17%; Table 2). According to our results, 83% (n=29) of the participants found this method of learning, including the small group size and the method of facilitation (combining both PCPs and specialists), as the most appropriate.

### Program Satisfaction and Improved Confidence

Overall, a high level of satisfaction with the provided materials was reported among respondents (29/35, 83%). Moreover, 89% (31/35) indicated that they would likely recommend the program to others, although only 57% (20/35) found the platform easy to use. A higher percentage (9/11, 75%) of the participants in cycle 4 reported easy navigation of the activity compared to participants in cycle 1 (3/10, 30%; Table 2).

**Figure 1.**
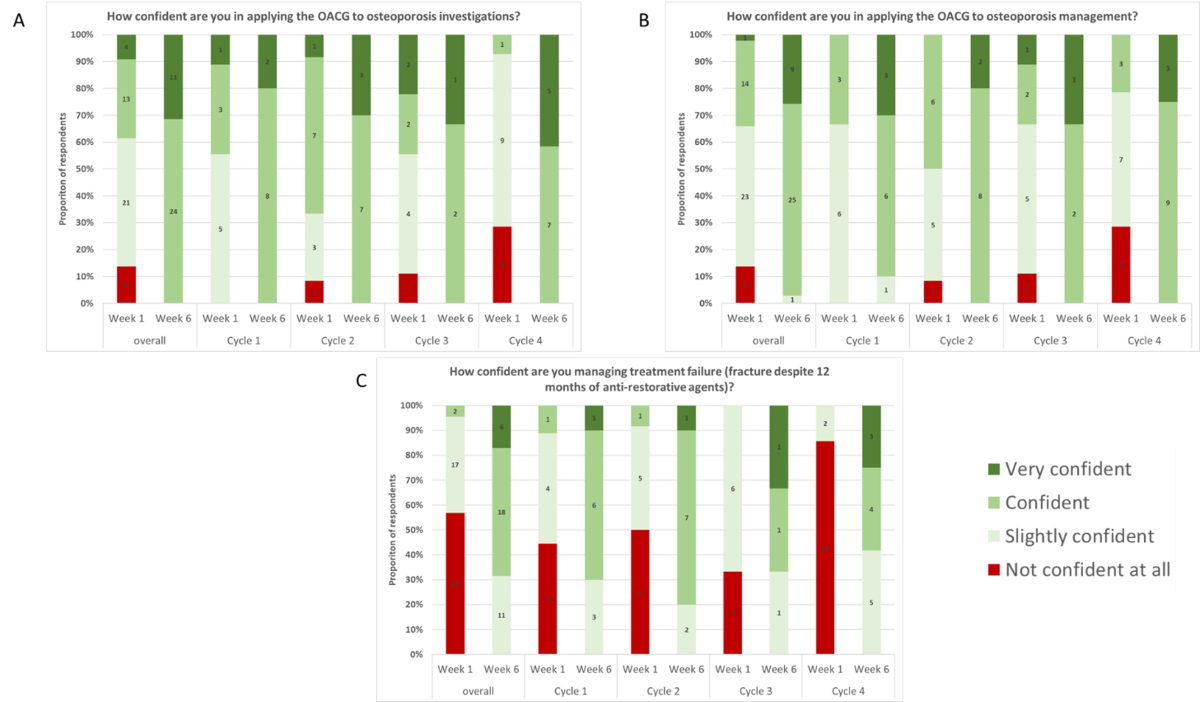
Change in primary care physicians’ (PCPs) confidence in osteoporosis at the end of the learning cycle. OACG: Osteoporosis Australia Clinical Guidelines.

At week 1, 34% (n=10) of the PCPs were confident or very confident in applying the current guidelines of managing patients, and less than 40% (n=11) were confident or very confident in applying the current guidelines for osteoporosis investigation. Following the course completion, almost everyone reported being confident or very confident in these topics (Figure 1).

### Quiz Results or Level of Knowledge or Self-Assurance Gain

The average marks for the individual module-related quiz questions were 70%, ranging from 22% to 100% (Figure S1 and Table S2 in Multimedia Appendix 1).

The results show that 98%-100% (n=34-35) of the participants provided correct answers to osteoporosis risk factors and pharmacological treatment (questions 10-17), while 22%-33% (n=8-12) of the participants provided correct responses to osteoporosis therapy−related questions (questions 37-39; Figure S1 in Multimedia Appendix 1).

Levels of participants’ postprogram confidence are demonstrated in Figures1  2 and Table S1 in Multimedia Appendix 1. The results show that 86% (n=24) of the respondents rated the activity as entirely relevant to their practice. Additionally, 68% (n=19) of the respondents reported that they were able to design a structured procedure for monitoring osteoporosis.

**Figure 2.**
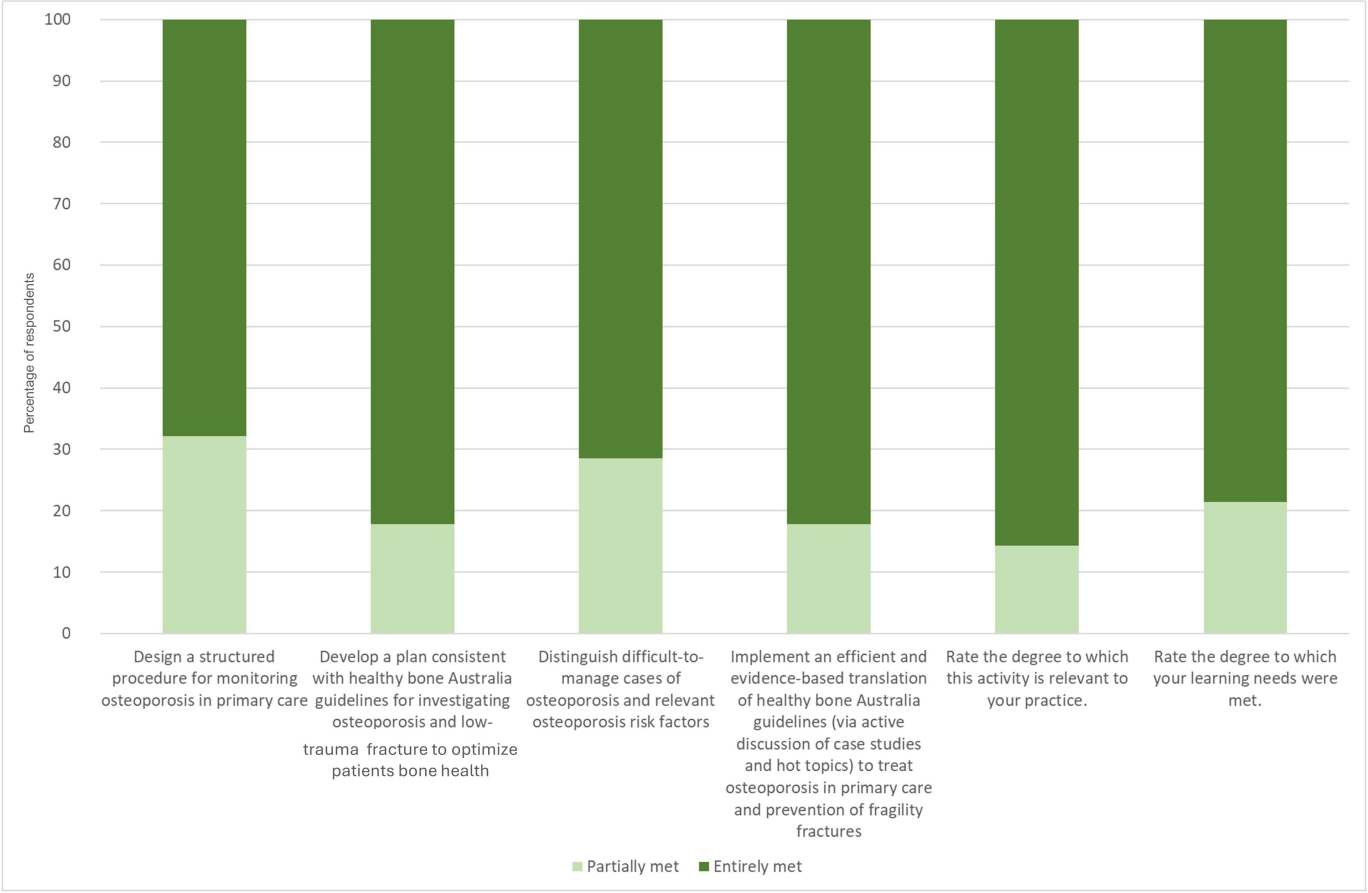
Postprogram self-assurance level of participants.

## Discussion

### Principal Findings

Our findings demonstrate that the participating PCPs reported a considerable boost in their knowledge and confidence in osteoporosis management in primary care. While hospital-centered FLSs are cost-effective for secondary fracture prevention [7,37], their expansion in Australia is projected to have a limited impact on reducing minimal trauma fractures in older adults, with high associated costs [38], necessitating alternative strategies. Many patients rely on PCPs for postfracture care [39], underscoring the need for updated guideline adherence. However, global and Australian underuse of osteoporosis guidelines in primary care persists, leading to underdiagnosis and undertreatment [7,37,40-43]. Furthermore, a considerable proportion of Australians lack timely access to osteoporosis care in primary health care settings, with studies demonstrating low treatment rates for those meeting intervention criteria [16,39,44]. For instance, 2 Australian studies revealed that only 30% of the postmenopausal women with fractures received osteoporosis therapy [9], and 76% of the fractured men remained untreated for 1 year postfracture [16]. The CFC Learning Hub, a tailored VCoP, was developed to address these disparities through evidence-based education and peer collaboration.

PCPs face daily challenges in delivering optimal care for patients with chronic health issues, requiring evidence-based treatment for the best outcomes [29,31]. VCoPs for CPD can help overcome barriers such as time limitations and preferences for in-person learning [23]. Despite their potential to enhance PCPs’ knowledge and skills, challenges remain in their effective use for CPD. Our team identified 7 key areas vital for crafting and maintaining efficient VCoPs, including distrust of online information, accommodating individual learning preferences, and bridging communication gaps between PCPs and specialists [31]. Based on these insights, our pilot program developed the CFC Learning Hub to enhance PCPs’ understanding of osteoporosis management, supporting the implementation of evidence-based guidelines [29].

The CFC pilot program identified osteoporosis treatment and prevention strategies, as well as risk assessment, as the topics initiating the most discussion, indicating an interest in these areas. Thus, the CFC Learning Hub program’s topic modules focused on osteoporosis, treatment, monitoring, and challenging conditions. The program received high satisfaction rates in that about 83% (n=29) of the program evaluation respondents were satisfied with the content, most respondents were either entirely (n=22, 73%) or partly (n=6, 23%) satisfied that their training needs had been met, and 89% (n=31) expressed that they were likely to recommend the training, reflecting the program’s ability to instill confidence in the PCP participants. It is pinpointed that efficient VCoPs depend on knowledge exchange among members, which can be implemented in clinical settings [45]. Health professionals showed greater engagement when VCoPs focused on patient-centered approaches, emphasizing patient needs, concerns, and preferences [46]. Overall, in terms of structure, it appears that our project successfully crafted a program that fulfills PCPs’ requirements for an interactive curriculum with practical, relevant content. Moreover, for VCoPs seeking to foster peer-to-peer interaction and enhance program satisfaction, it is advisable to design their content and modules incorporating clinically relevant practice-based cases.

Particularly since the COVID-19 pandemic, the workload of PCPs has significantly increased, stretching their time commitments and exacerbating their isolation from peers [47,48]. VCoPs leveraging technology offer opportunities for learning, collaboration, and information exchange, overcoming barriers like geography, cost, and time zone differences [21,25-27]. However, time remains a major barrier to participation in VCoPs [49,50], with flexibility in scheduling being critical for engagement. Compared to timed webinars and their fixed question times, the trend of high popularity of participation during evenings (n=23, 66%) and weekends (n=10, 29%) in our learning activity underscores its ability to deliver the needed flexibility, catering to participants’ preferences for accessing content outside traditional work hours, with fewer opting for weekdays (n=2, 5.7%). Further, analyzing the preferred access times of PCPs for the program enabled us to pinpoint optimal communication windows, ensuring maximum engagement during peak activity periods and tailoring our outreach efforts accordingly. This aligns with evidence that adaptable VCoPs mitigate time barriers [27]. Overall, a VCoP tailored for PCPs' professional education must cater to their demanding schedules, offering flexibility for participation during off-hours such as evenings and weekends. In addition to scheduling flexibility, ease of use is vital for VCoP effectiveness. For a virtual community to thrive, participants must possess a basic level of technical proficiency in using information and communication technologies [51]. However, mastering e-communication tools can impose a steep learning curve, demanding time from already busy health professionals [52]. Some studies highlight a deficiency in health professionals’ competence with the technology necessary for effective VCoP engagement [50]. For instance, a study exploring an online platform for professional development found that nurses and other health professionals, despite considering themselves “computer literate,” required substantial mentoring and support in the virtual environment [53]. In our activity, just over half (n=20, 57%) of the participants reported finding the platform user-friendly, with the majority of access problems reported in the first two cycles of the 4 cycles conducted. To address PCPs’ unpreparedness with platform use, we implemented an induction session with a video recording available throughout the 6-week activity, which led to a significant decrease in access issues in later cycles. Proactive communication during registration and before the activity further familiarized participants with the platform, nearly eliminating complaints about usability. This supports the idea that technical skills for VCoPs should be developed during prequalifying education to ensure a solid foundation for virtual collaboration in clinical practice [54]. Indeed, it is argued that to ensure interprofessional teams can collaborate effectively, practicing professionals are required to communicate in both face-to-face and virtual environments [54]. Hence, the availability of preactivity induction tools on platform navigation and functionality could enhance participation rates and mitigate potential nonparticipation among PCPs due to technical limitations.

A primary motivation behind online communities is to establish networks of individuals sharing common interests, despite being geographically distant [21]. Virtual communities thrive when there is a collective eagerness to exchange knowledge and experiences; however, without community-driven engagement, participation tends to be sporadic and restricted [55]. To foster trust and cohesion, our program specifically recruited PCPs, excluding other health care practitioners. Also, recruitment advertisements emphasized the activity’s focus on bone health advancements, attracting participants with shared interests, particularly the desire to improve patient bone health management (a motivation nominated by 24, 69% of the respondents). It is indicated that VCoPs with a specialized focus tend to attract more engaged members [45]. Additionally, our program was free, aligning with the understanding that health care practitioners engage more when resources are accessible and low-cost [46]. Besides targeting homogenous groups of members and ensuring the program’s affordability, group size also influences engagement; smaller groups (12‐20 members) enhance participation and learning [34,56,57]. Our four 6-week cycles involved 55 PCPs (average of 13‐14 per cycle), fostering a sense of community. Studies show that small VCoPs reduce isolation and increase knowledge sharing [34]. Similarly, most participants in our CFC Learning Hub indicated that the size of their groups was suitable for their educational experience. Finally, VCoP’s facilitators play a key role in maintaining engagement by setting clear rules, focusing discussions, and promoting respect [21]. Learning from experts (n=8, 23%) ranked as the second-highest motivation for joining the activity. In the CFC Learning Hub, experienced PCP peers led discussions, supported by specialists to guide case analyses and foster engagement. Facilitators’ detailed profiles and access to support ensured high satisfaction and participation. All in all, the management of participation issues related to VCoPs, such as sense of community, affordability, and proper facilitation, may contribute to increased active participation and engagement.

Health care VCoPs use various digital formats, including teleconferences, webinars, videoconferences, online meeting spaces, websites, emails, intranets, and social media [58]. Some also feature blogs, online discussion forums, or file repositories [59]. One example is Project Extension for Community Healthcare Outcomes, using real-time videoconferencing to connect rural primary care providers with specialists [32], resulting in significant improvements in provider confidence and addressing treatment gaps through education and knowledge-sharing. Social media platforms like Facebook, X (formerly known as Twitter), and LinkedIn can also host VCoPs [27]. The diversity in digital formats among VCoPs reflects the diverse preferences and interests of health professionals, highlighting the absence of a one-size-fits-all format. Similarly, such variation in interests was demonstrated in our program. The study’s results indicated varying preferences among participants regarding their favored learning models, with a significant proportion (n=13, 37%) endorsing small group learning, followed by live webinars (n=9, 26%), interactive learning methods (n=7, 20%), and on-demand videos (n=6, 17%). Clearly, a small group online VCoP such as our program must leverage various digital formats to effectively disseminate information and knowledge, catering to the diverse learning preferences of participants. For instance, based on feedback received in earlier cycles, we proceeded to incorporate additional knowledge-sharing formats, such as the introduction of short videos to the modules. Overall, a successful health VCoP must adapt to the diverse preferences in learning models among health professionals. Therefore, it may be advantageous to use multiple learning formats to ensure broad participation among participants.

PCPs play a crucial role in managing fragility fractures, highlighting the importance of equipping them with adequate resources and knowledge. However, PCPs globally, including in Australia, demonstrate low-level adoption of evidence-based osteoporosis management guidelines, leading to underdiagnosis and undertreatment. Our program aimed to address these gaps by developing an interactive online educational platform, the CFC Learning Hub, targeting PCPs to enhance their understanding of osteoporosis management models. The program’s success was evidenced by high participant satisfaction rates and high engagement levels. This success was supported by our program implementing strategies to avoid time barriers (including allowing flexible times of engagement), reduce access-related issues, and enhance participant engagement. Overall, our educational initiative transforms the learning experience by offering flexible time arrangements, interactive peer collaboration, and detailed insights into osteoporosis management; tailoring to various patient demographics; and facilitating practice-oriented learning for PCPs.

### Limitations

The time data were based on the time between clicks and may not accurately represent the time spent by PCPs on learning or accessing educational materials. Specifically, it was not possible to differentiate between the use of a single or dual screen or switching between different documents once opened or downloaded. While this is not a live-video interaction, it is possible that the participants opened the website without actually learning. Therefore, the true duration of the learning cannot be confirmed. Additionally, participants were recruited via the Praxhub platform, and the vast majority of them were practicing in metropolitan areas and had over 10 years of practice experience. Therefore, our results might not be transferable to all practitioners, particularly targeting regional PCP practices and younger practitioners. Third, participants were not asked to complete a knowledge quiz prior to the training, which could lead to an overestimation of the effect of the intervention on improved knowledge.

### Conclusions

Addressing the treatment gap in osteoporosis care, especially within primary care, where PCPs are pivotal, is urgently needed. Challenges like low trust of online information sources, communication gaps, and inadequate patient information sharing highlight the necessity for tailored educational interventions like the CFC Learning Hub. The program, characterized by its user-friendly, case-based, interactive, and time-flexible nature, effectively bridged investigation and management gaps in osteoporosis. Specifically designed to tackle challenges encountered by PCPs, this platform holds promise for professional development across various health-related fields.

## Supplementary material

10.2196/76216 Multimedia Appendix 1 Quiz performance, postprogram participant feedback, and quiz item details.

## Abbreviations

CFC: Community Fracture Capture
CPD: continuing professional development
FLS: fracture liaison service
PCP: primary care physician
VCoP: virtual community of practice
