# Supplementary material for: Evaluation of a Novel Web-Based Active Learning Tool for Primary Care Physicians’ Continuing Professional Development (The Community Fracture Capture Learning Hub): Quantitative Analysis
Source: JMIR Form Res. 2026 Jan 21;10:e76216. doi: 10.2196/76216 (PMC12822860; doi:10.2196/76216)
Supplement: Multimedia Appendix 1 [file formative-v10-e76216-s001.docx]

**Supplementary Figures:**

Figure S1 Quiz - percentage of correct responses per each question


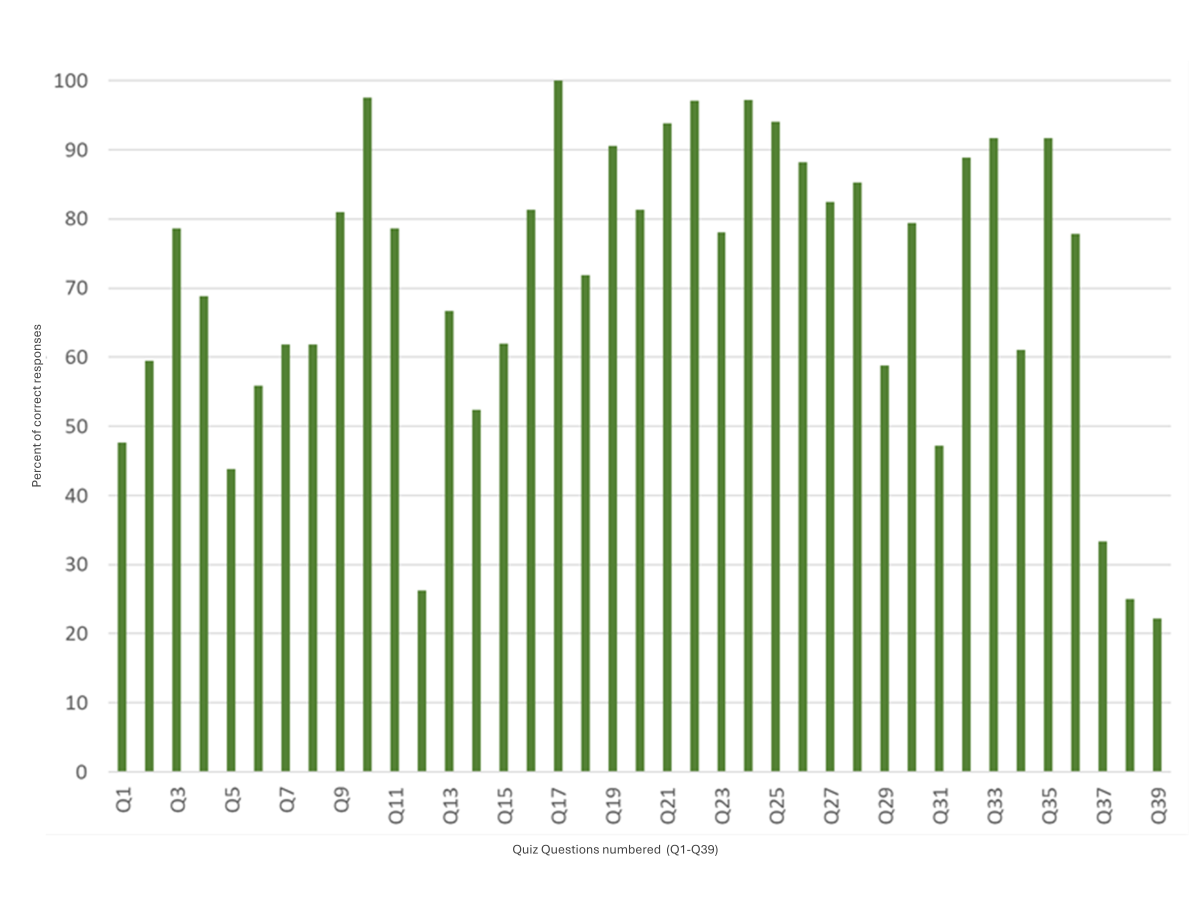


**Supplementary tables:**

Table S1 Meeting Participants Expectations Post Program (At week 6)

|  | Total | | Cycle 1 | | Cycle 2 | | Cycle 3 | | Cycle 4 | | |  |
| --- | --- | --- | --- | --- | --- | --- | --- | --- | --- | --- | --- | --- |
|  | n | % | n | % | n | % | n | % | n | | % |  |
| Overall responses | 28 |  | 6 |  | 9 |  | 2 |  | 11 | |  |  |
| Design a structured procedure for monitoring osteoporosis in primary care | | | | | | | | | | | |  |
| Entirely met | 19 | 67.9 | 5 | 83 | 6 | 67 | 2 | 100 | 6 | | 55 |  |
| Partially met | 9 | 32.1 | 1 | 17 | 3 | 33 | 0 | 0 | 5 | | 45 |  |
|  |  |  |  |  |  |  |  |  |  | |  |  |
| Develop a plan consistent with healthy bone Australia guidelines for investigating osteoporosis and low-trauma fracture to optimise patients bone health | | | | | | | | | | | |  |
| Entirely met | 23 | 82.1 | 4 | 67 | 8 | 89 | 2 | 100 | 9 | | 82 |  |
| Partially met | 5 | 17.9 | 2 | 33 | 1 | 11 | 0 | 0 | 2 | | 18 |  |
|  |  |  |  |  |  |  |  |  |  | |  |  |
| Distinguish difficult-to-manage cases of osteoporosis and relevant osteoporosis risk factors | | | | | | | | | | | |  |
| Entirely met | 20 | 71.4 | 5 | 83 | 5 | 56 | 2 | 100 | 8 | | 73 |  |
| Partially met | 8 | 28.6 | 1 | 17 | 4 | 44 | 0 | 0 | 3 | | 27 |  |
|  |  |  |  |  |  |  |  |  |  | |  |  |
| Implement an efficient and evidence-based translation of healthy bone Australia guidelines (via active discussion of case studies and hot topics) to treat osteoporosis in primary care and prevention of fragility fractures | | | | | | | | | | | |  |
| Entirely met | 23 | 82.1 | 5 | 83 | 6 | 67 | 2 | 100 | 10 | | 91 |  |
| Partially met | 5 | 17.9 | 1 | 17 | 3 | 33 | 0 | 0 | 1 | | 9 |  |
|  |  |  |  |  |  |  |  |  |  | |  |  |
| Rate the degree to which this activity is relevant to your practice. | | | | | | | | | | | |  |
| Entirely met | 24 | 85.7 | 5 | 83 | 8 | 89 | 2 | 100 | 9 | | 82 |  |
| Partially met | 4 | 14.3 | 1 | 17 | 1 | 11 | 0 | 0 | 2 | | 18 |  |
|  |  |  |  |  |  |  |  |  |  |  | | |
| Rate the degree to which your learning needs were met. | | | | | | | | | | | |  |
| Entirely met | 22 | 78.6 | 5 | 83 | 6 | 67 | 2 | 100.0 | 9 | | 82 |  |
| Partially met | 6 | 21.4 | 1 | 17 | 3 | 33 | 0 | 0.0 | 2 | | 18 |  |

Table S2: Quiz Questions ID and the Percentage of Correct Responses

| Question ID | % of correct responses | | | | | | |
| --- | --- | --- | --- | --- | --- | --- | --- |
|  | | Overall | Cycle 1 | Cycle 2 | Cycle 3 | Cycle 4 |  |
| Q1 | | 47.6 | 41.7 | 41.7 | 60.0 | 53.8 |  |
| Q2 | | 59.5 | 83.3 | 58.3 | 60.0 | 38.5 |  |
| Q3 | | 78.6 | 91.7 | 83.3 | 60.0 | 69.2 |  |
| Q4 | | 68.8 | 77.8 | 77.8 | 40.0 | 66.7 |  |
| Q5 | | 43.8 | 55.6 | 55.6 | 0.0 | 44.4 |  |
| Q6 | | 55.9 | 66.7 | 45.5 | 75.0 | 50.0 |  |
| Q7 | | 61.8 | 88.9 | 63.6 | 50.0 | 40.0 |  |
| Q8 | | 61.8 | 77.8 | 54.5 | 25.0 | 70.0 |  |
| Q9 | | 81.0 | 66.7 | 91.7 | 100.0 | 76.9 |  |
| Q10 | | 97.6 | 100.0 | 91.7 | 100.0 | 100.0 |  |
| Q11 | | 78.6 | 75.0 | 75.0 | 100.0 | 76.9 |  |
| Q12 | | 26.2 | 25.0 | 16.7 | 0.0 | 46.2 |  |
| Q13 | | 66.7 | 66.7 | 75.0 | 40.0 | 69.2 |  |
| Q14 | | 52.4 | 41.7 | 66.7 | 0.0 | 69.2 |  |
| Q15 | | 61.9 | 50.0 | 58.3 | 60.0 | 76.9 |  |
| Q16 | | 81.3 | 77.8 | 77.8 | 80.0 | 88.9 |  |
| Q17 | | 100.0 | 100.0 | 100.0 | 100.0 | 100.0 |  |
| Q18 | | 71.9 | 77.8 | 77.8 | 40.0 | 77.8 |  |
| Q19 | | 90.6 | 88.9 | 100.0 | 60.0 | 100.0 |  |
| Q20 | | 81.3 | 88.9 | 66.7 | 100.0 | 77.8 |  |
| Q21 | | 93.8 | 100.0 | 100.0 | 100.0 | 77.8 |  |
| Q22 | | 97.1 | 88.9 | 100.0 | 100.0 | 100.0 |  |
| Q23* | | 78.1 | 88.9 | 66.7 | 80.0 | 77.8 |  |
| Q24 | | 97.2 | 100.0 | 100.0 | 100.0 | 92.3 |  |
| Q25 | | 94.1 | 100.0 | 90.9 | 100.0 | 90.0 |  |
| Q26 | | 88.2 | 77.8 | 90.9 | 100.0 | 90.0 |  |
| Q27 | | 82.4 | 77.8 | 72.7 | 75.0 | 100.0 |  |
| Q28 | | 85.3 | 88.9 | 90.9 | 75.0 | 80.0 |  |
| Q29 | | 58.8 | 55.6 | 36.4 | 100.0 | 70.0 |  |
| Q30 | | 79.4 | 77.8 | 81.8 | 50.0 | 90.0 |  |
| Q31 | | 47.2 | 37.5 | 66.7 | 0.0 | 46.2 |  |
| Q32 | | 88.9 | 87.5 | 100.0 | 100.0 | 76.9 |  |
| Q33 | | 91.7 | 100.0 | 91.7 | 66.7 | 92.3 |  |
| Q34 | | 61.1 | 87.5 | 50.0 | 33.3 | 61.5 |  |
| Q35 | | 91.7 | 87.5 | 91.7 | 100.0 | 92.3 |  |
| Q36 | | 77.8 | 87.5 | 75.0 | 66.7 | 76.9 |  |
| Q37 | | 33.3 | 50.0 | 33.3 | 66.7 | 15.4 |  |
| Q38 | | 25.0 | 50.0 | 33.3 | 0.0 | 7.7 |  |
| Q39 | | 22.2 | 25.0 | 25.0 | 33.3 | 15.4 |  |

Note: * the Australian Pharmaceutical Benefits Scheme (PBS) indications for romosozumab changed from 1/11/2024.

Table S3: Quiz Questions Content

| **Question ID** | **Question** |
| --- | --- |
| Q1 | **Approximately what proportion of older people who are found to have a vertebral compression fracture do not report a history of significant acute back pain?** |
| Q2 | **A history of low-trauma hip fracture in a parent is associated with how much approximate increase in a person’s risk of hip fracture?** |
| Q3 | **Which of the following forms of diabetes are associated with an increased fracture risk?** |
| Q4 | **With regard to osteoporosis diagnosis and monitoring, which of the following is true?** |
| Q5 | **Which of the following is true regarding the management of osteoporosis in the elderly?** |
| Q6 | **Which of the following is not true regarding osteonecrosis of the jaw (ONJ)?** |
| Q7 | **Which of the following is a major criterion for the diagnosis of atypical femoral fractures (AFF)?** |
| Q8 | **Which of the following is not true regarding atypical femoral fractures (AFF)?** |
| Q9 | **Which of the following forms of physical activity has the best evidence for improving fall-related outcomes in older people?** |
| Q10 | **Which of the following is a common modifiable risk factor for osteoporosis and/or fractures?** |
| Q11 | **Which of the following conditions is potentially associated with secondary osteoporosis?** |
| Q12 | **Among patients presenting with a low-trauma fracture consistent with osteoporosis, approximately what proportion will have osteoporosis based on their T-score found on bone density testing?** |
| Q13 | **Which of the following is the best indicator of current vitamin D nutritional status?** |
| Q14 | **Research indicates that calcium supplementation in postmenopausal women reduces low-trauma fracture risk by how much on average?** |
| Q15 | **Calcium supplementation usually is recommended in patients being treated with specific medications for osteoporosis mainly for the following reason** |
| Q16 | **Which of the following is not recommended for general bone health maintenance and fracture prevention in all patients?** |
| Q17 | **The choice of pharmacological therapy for osteoporosis should be based upon?** |
| Q18 | **Which of the following is true regarding pharmacologic approaches to prevention and treatment of osteoporosis?** |
| Q19 | **Therapeutic treatment pause should not be considered for which of the following agent(s)?** |
| Q20 | **In which of the following scenarios does antiresorptive therapy not have strong evidence for efficacy?** |
| Q21 | **In adult patients with osteoporosis treated with denosumab, administration of denosumab should not be delayed or stopped without subsequent antiresorptive therapy to decrease the risk of?** |
| Q22 | **Risk factors for developing hypocalcaemia after denosumab therapy include which of the following** |
| Q23 | **Anabolic therapies such as teriparatide and romosozumab can be prescribed using the PBS in the following circumstances** ***** |
| Q24 | **Which of the following is a criterion for PBS-subsidised prescription of teriparatide?** |
| Q25 | **Oral bisphosphonates should not be used as initial therapy in patients with:** |
| Q26 | **After starting and antiresorptive agent the optimal timing of densitometry scans is:** |
| Q27 | **Bisphosphonate treatment holidays can be considered in which of the following circumstances** |
| Q28 | **After a bisphosphonate treatment holiday has begun, fracture risk and BMD should be re-evaluated** |
| Q29 | **Which of the following is false regarding the acute-phase reaction (flu-like symptoms, e.g pyrexia and myalgia) that can occur after IV zoledronic acid** |
| Q30 | **Regarding bisphosphonate-associated atypical femoral fractures (AFF), which of the following is true:** |
| Q31 | **Definition of anti-resorptive treatment failure includes?** |
| Q32 | **Which of the following can contribute to proven treatment failure?** |
| Q33 | **What is the usual frequency of injections for romosozumab therapy?** |
| Q34 | **Which of the following is the most frequent adverse effect of romosozumab therapy?** |
| Q35 | **Which of these agents, when discontinued and no alternative anti-resorptive or anabolic bone therapy is commenced, results in significant loss of therapy-related bone mineral density gains within the first 12 months of cessation?** |
| Q36 | **In patients established on denosumab therapy who are then given a single dose of zoledronic acid in order to transition off Denosumab, which of the following is true** |
| Q37 | **Following denosumab administration for 24 months then teriparatide administration for a further 24 months, at which site is bone density reduced at the end of the 48 months compared with bone mineral density assessed after the 24 months of denosumab?** |
| Q38 | **Which of the following treatment sequences will result in the greatest BMD rise?** |
| Q39 | **In postmenopausal women treated with zoledronic acid for 3 years then placebo for 3 years compared with those treated with zoledronic acid for 6 years, at which site is bone mineral density reduced by the greatest amount at the 6-year assessment?** |

Note: Q1 to Q10, Q11 to Q20, Q21 to Q30, and Q31 to Q39 were included in weeks 2, 3, 4 and 5, respectively.
